# Intrinsic Motivation Inventory for Heart‐Healthy Lifestyle (IMI‐HeartLife) in Cardiovascular Disease Prevention: A Validation Study

**DOI:** 10.1002/nop2.70406

**Published:** 2025-12-23

**Authors:** Jina Choo, Songwhi Noh, Yura Shin

**Affiliations:** ^1^ College of Nursing Korea University Seoul South Korea; ^2^ Transdisciplinary Major in Learning Health Systems Graduate School, Korea University Seoul South Korea

**Keywords:** cardiovascular diseases, health behaviour, healthy lifestyle, motivation, primary prevention, psychometrics

## Abstract

**Aim:**

To validate a modified version of the Intrinsic Motivation Inventory (IMI)—the IMI‐HeartLife—adapted for heart‐healthy lifestyle behaviours in community‐dwelling adults without a history of cardiovascular disease. While the original IMI has been validated in contexts such as competitive sports and education, its use in healthcare—particularly for cardiovascular disease prevention—has been limited.

**Design:**

A validation study addressing content and construct validity and reliability.

**Methods:**

The IMI‐HeartLife was refined through three phases: (1) conceptualisation and item pool generation, (2) evaluation of content and face validity and (3) psychometric evaluation. As part of the psychometric evaluation, two established instruments were employed to assess the discriminant and convergent validity of the IMI‐HeartLife scale.

**Results:**

A total of 703 community‐dwelling adults were recruited and randomly assigned to two subsamples for exploratory (*n* = 352) and confirmatory (*n* = 351) factor analyses. From the initial 32 items drawn from five original IMI subscales, 16 were retained. A three‐factor structure—Interest/Enjoyment, Choice/Value/Usefulness and Competence/Effort—was supported (CMIN/DF = 3.43, SRMR = 0.06, RMSEA = 0.08, CFI = 0.95, TLI = 0.94). Discriminant and convergent validity of the IMI‐HeartLife were confirmed, and internal consistency and test–retest reliability were acceptable.

**Conclusions:**

The 16‐item IMI‐HeartLife demonstrated good validity and reliability as a measure of intrinsic motivation to promote heart‐healthy lifestyle in cardiovascular disease prevention.

**Implications for the Profession:**

In community and primary care settings, nurses are well positioned to assess intrinsic motivation as a core cognitive construct and to deliver personalised interventions that promote heart‐healthy lifestyle behaviours in the context of cardiovascular disease prevention. Within this framework, the IMI‐HeartLife may serve as a valuable instrument for assessing intrinsic motivation and guiding individualised nursing strategies.

**Reporting Method:**

COSMIN reporting guideline for studies on measurement properties of patient‐reported outcome measures (PROM): version 2.0.

**Public Engagement:**

The content analysis was conducted in collaboration with an interdisciplinary user group including eight healthcare professionals.

## 1 Introduction

Cardiovascular disease is largely preventable through the adoption of comprehensive heart‐healthy lifestyle behaviours, such as smoking cessation, avoidance of high‐risk alcohol consumption, adherence to a healthy diet, regular physical activity and maintenance of a healthy body weight (Ahmadi et al. 2023; Tsai et al. 2021; Wu et al. 2024). Given the critical importance of these lifestyle behaviours in cardiovascular prevention, nurses are uniquely positioned to lead such efforts in clinical and community settings. In this regard, the Preventive Cardiovascular Nurses Association emphasises the essential role of nurses in encouraging sustainable behavioural changes to improve heart health and general wellness (Preventive Cardiovascular Nurses Association 2025). Meanwhile, advances in cardiovascular disease prevention have been driven by the identification of modifiable behavioural factors, which in turn have informed evidence‐based behavioural recommendations and interventional strategies to facilitate heart‐healthy behaviour change in clinical contexts (Stuart‐Shor et al. 2012). Nevertheless, in practical settings, such interventions often remain predominantly informational, lacking the behavioural components necessary to promote the integration, long‐term sustainability and internalisation of heart‐healthy behaviours—particularly in primary care and community‐based settings.

Heart‐healthy behavioural interventions, initially developed for use in cardiac rehabilitation settings (Redfern et al. 2022), have progressively expanded into primary care and community‐based settings at both national and metropolitan levels (Choo et al. 2021; Laddu et al. 2021). As cardiovascular prevention efforts continue to scale, there is an increasing need for nurses to acquire the competencies necessary to facilitate both the initiation and sustained practice of heart‐healthy behaviours. Within this context, motivational interviewing (Mifsud et al. 2020) and motivational communication techniques (Duffy et al. 2021; Xu et al. 2023) have gained prominence, underscoring the value of interactive, person‐centred approaches. Specifically, motivational interviewing, in particular, fosters sustained engagement in desirable behaviours through individualised dialogue that takes into account the person's unique psychosocial background (Miller and Rollnick 1991), and has demonstrated efficacy in behavioural changes of smoking cessation and physical activity (Frost et al. 2018; Soria et al. 2006). Reportedly, nurse‐led community‐based motivational interviewing was significantly effective on changes in composite scores of multiple heart‐healthy behaviours among individuals without a history of cardiovascular disease (Choo et al. 2023a, 2023b). Against this background, nurses need to apply motivation‐enhancing strategies in practice to facilitate and promote changes in heart‐healthy behaviours within the context of cardiovascular disease prevention. To maximise the effectiveness of such practical applications, it is essential to first assess individuals' motivation for behaviour change and then develop tailored strategies based on their motivational level. Accordingly, the assessment of motivation prior to intervention serves as a critical foundation for designing context‐specific strategies to promote heart‐healthy behaviours in cardiovascular disease prevention.

Motivation has traditionally been conceptualised as the degree of an individual's need or drive for competence and self‐determination in human behaviours—particularly intrinsic motivation, as conceptualised by Self‐Determination Theory (Center for Self‐Determination Theory 2025; Deci and Ryan 2013). Intrinsically motivated behaviours are sustained when individuals perceive external barriers as controllable and when their actions are aligned with personal values and integrated aspects of the self (Deci and Ryan 2013). Within this framework, motivation is situated along a continuum of self‐determination, ranging from amotivation to intrinsic motivation. In parallel, the Information‐Motivation‐Behavioural Skills (IMB) model explicitly incorporates motivation as a core construct (Fisher et al. 2003), positing that it exerts both a direct effect on the adoption of health behaviours and an indirect effect on facilitating health behaviours via the development of behavioural skills (Fisher et al. 2003). This suggests that motivation, as a cognitive construct, is considered a powerful antecedent of health behaviour adoption.

Ryan (1982) and his colleagues (Plant and Ryan 1985; Ryan et al. 1983) developed the Intrinsic Motivation Inventory (IMI) comprising seven subscales of Interest/Enjoyment, Perceived competence, Effort/Importance, Pressure/Tension, Perceived choice, Value/Usefulness and Relatedness (McAuley et al. 1989). As a generic measure, the psychometric properties of the IMI has been validated in specific contexts such as competitive sports (McAuley et al. 1989) and academic learning environments (Ostrow and Heffernan 2018). However, its application in healthcare contexts remains limited, specifically in the context of heart‐healthy lifestyle for cardiovascular disease prevention. Accordingly, it is imperative to validate the psychometric properties of the IMI in the context of heart‐healthy behaviours to enable its practical application and broader utilisation in nursing practice.

## 2 The Study

### 2.1 Aims

The present study aims to adapt the original IMI to the context of heart‐healthy lifestyle behaviours for the prevention of cardiovascular disease, and to evaluate the psychometric properties of the adapted instrument—termed the IMI for Heart‐Healthy Lifestyle (IMI‐HeartLife)—through a systematic validation process conducted among community‐dwelling adults with no history of cardiovascular disease.

## 3 Methods

**FIGURE 1:**
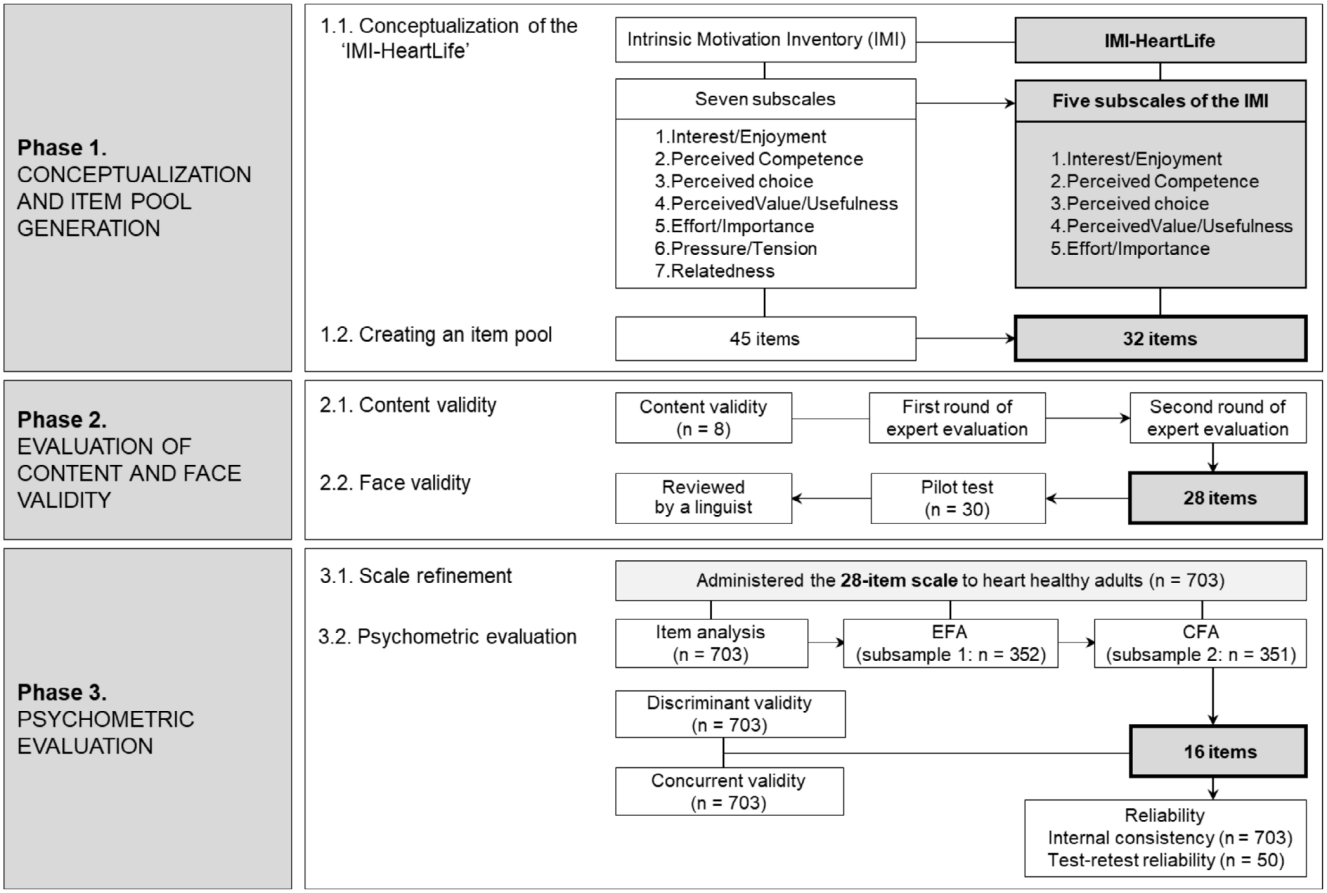
The study procedure for psychometric evaluation of the IMI‐HeartLife. IMI‐HeartLife, Intrinsic Motivation Inventory for Heart‐Healthy Lifestyle.

We carried out the study procedure according to established scale development guidelines in three phases (Celebi Cakiroglu and Baykal 2021; DeVellis 2016) (Figure 1): (1) conceptualisation and item pool generation, (2) evaluation of the content validity and face validity and (3) psychometric evaluation.

### 3.1 Phase 1: Conceptualisation and Item Pool Generation

The IMI‐HeartLife was conceptualised as an ‘internal drive that compels individuals to engage in and maintain healthy lifestyle behaviours beneficial to cardiovascular health’ based on the Self‐Determination Theory (Deci and Ryan 1985). An initial item pool of 32 items (Table S1) was created by reviewing the seven subscales of the original ‘Post‐Experimental IMI’ (Center for Self‐Determination Theory 2025).

Based on IMI guidance that subscales can be selectively employed according to the study's focus (Center for Self‐Determination Theory 2022), we selected five after excluding two subscales of Pressure/Tension and Relatedness. The two excluded subscales represent constructs that are theoretically and linguistically distinct from intrinsic motivation within the self‐regulation continuum of Self‐Determination Theory. Specifically, Pressure/Tension is conceptually associated with controlled or introjected regulation rather than autonomous motivation, while relatedness reflects a basic psychological need rather than an inherent source of enjoyment (Deci and Ryan 2000). This exclusion was therefore deemed appropriate, as the present study aimed to capture self‐directed, volitional engagement in heart‐healthy lifestyle behaviours guided by internalised forms of autonomous regulation, rather than externally imposed demands or relational dynamics. Moreover, items from the Pressure/Tension subscale (e.g., “I felt very tense while doing this activity”) appear more suitable for activities that inherently involve competition or evaluative stress, such as sports or performance tasks (Cocca et al. 2022; Gibbens 2019), rather than for health‐promoting lifestyle behaviours. Similarly, items from the Relatedness subscale (e.g., “I felt like I could really trust this person”) emphasise dyadic relationships with a specific individual (“this person”), which do not align with the broader social context in which heart‐healthy lifestyle behaviours are typically adopted—namely, behaviours driven by personal motivation, self‐discipline and general social support rather than singular interpersonal dynamics.

For the retained items, a seven‐point Likert scale ranging from 1 (very low agreement) to 7 (very high agreement) was employed (McAuley et al. 1989), and the original wording “this activity” was modified to “healthy lifestyle.” The questionnaire included an instructional statement defining “heart‐healthy lifestyle” behaviours as practices aimed at preventing cardiovascular disease. The IMI‐HeartLife items were originally developed in English and translated into Korean following standardised forward–backward translation procedures. Three nursing scholars, including the principal investigator—each with a doctoral degree earned in the United States and over 5 years of academic experience—independently conducted forward translations from English to Korean. The translated versions were reviewed during a consensus meeting to harmonise and consolidate item wording. The consolidated Korean version was then back‐translated into English by a professional translation agency. The back‐translated version underwent two additional rounds of expert review by the same nursing scholars to ensure semantic and conceptual equivalence. This was followed by a final linguistic review by a Korean language expert to confirm clarity and comprehensiveness after the pilot test for face validity, as described in the subsequent Phase 2.

### 3.2 Phase 2: Evaluation of the Content and Face Validity

Content validity was assessed through two rounds of expert evaluations, using the content validity index, including item‐level (I‐CVI) and scale‐level CVIs (S‐CVI) (Davis 1992). A panel of eight experts with backgrounds in scale development and cardiovascular health research—including one scale development expert, one cardiometabolic nursing specialist, four nursing scholars in heart‐healthy lifestyle modification, one cardiologist, and one exercise specialist with expertise in cardiovascular epidemiology—evaluated the items over two rounds. They rated the relevance and clarity of each item in relation to the construct of the IMI‐HeartLife using a four‐point Likert scale. In addition, the comprehensiveness of the items was assessed through open‐ended responses. Items were retained when the I‐CVI and S‐CVI exceeded thresholds of 0.80 and 0.90, respectively (Davis 1992). Face validity was evaluated through a pilot test in which the questionnaire, including open‐ended questions (Polit and Fm 2016), was administered to a sample of 30 heart‐healthy adults (Choo et al. 2023). The purpose was to assess the comprehensibility of the items—specifically, the use of jargon, reading level and overall clarity.

### 3.3 Phase 3: Psychometric Evaluation

First, item analysis was conducted to evaluate the extent to which each item represented the construct of the IMI‐HeartLife. Second, exploratory factor analysis (EFA) was performed to uncover the underlying factor structure of the item set. Third, confirmatory factor analysis (CFA) was carried out to verify the most plausible factorial structure identified during the exploratory phase. The discriminant and convergent validity and reliability of the ‘IMI‐HeartLife’ were examined. Finally, the measurement invariance of the IMI‐HeartLife was evaluated.

#### 3.3.1 Study Design and Participants

In Phase 3, a cross‐sectional, correlational study was conducted with 703 community‐dwelling adults. Participants were recruited through an accredited survey agency, Macromill Embrain Co. Ltd. Inclusion criteria were adults aged 19–64 years residing in South Korea. Exclusion criteria included a history of cardiovascular disease, mental health, physical disabilities, or cognitive impairments. Data were collected in February 2023, using a self‐administered online questionnaire delivered via a survey interface.

The minimum sample size was 640 (Garson 2008). A total of 703 participants were recruited, accounting for a dropout rate of 10%. No participants withdrew from the study. Participants were randomly divided into Subsample 1 (*n* = 352) for EFA and Subsample 2 (*n* = 351) for CFA. To assess test–retest reliability, 50 participants were randomly selected from the total sample (*N* = 703). Comparisons between the sample (*n* = 50) and the total sample showed no significant differences in general characteristics, confirming the representativeness of the sample (*n* = 50) (Table S2). The IMI‐HeartLife was re‐administered to these participants after a 15‐day interval (Terwee et al. 2007). This interval was deemed sufficient to minimise recall bias while preventing potential changes in cognitive recall that could arise with longer retest periods (Kim et al. 2015; Terwee et al. 2007). A retest period of approximately 2 weeks is widely used in psychometric reliability studies of self‐report instruments and is considered appropriate for assessing temporal stability while maintaining participant retention.

#### 3.3.2 Measures

The Situational Motivation Scale (SIMS) was used for the discriminant validity (Guay et al. 2000). The SIMS was developed and validated by Guay et al. (2000). It comprises four subscales—intrinsic motivation (4 items), identified regulation (4 items), external regulation (4 items) and amotivation (4 items)—resulting in a total of 16 items rated on a 7‐point Likert scale. Hwaing and Kim (2013) validated a Korean version of the SIMS, reducing the scale to 14 items. We utilised the Korean version of the SIMS. Subscale scores were computed as the mean of the items, with higher scores reflecting stronger endorsement of the corresponding subscales of the SIMS. The Cronbach's *α* of the scale ranged from 0.80 to 0.87 in the previous study (Hwaing and Kim 2013), and 0.56 to 0.94 in this study.

The Evaluation Tool for Metabolic Syndrome Modification Lifestyles was used for the convergent validity. The tool was developed and validated by Kang (2010) as a scale for assessing comprehensive lifestyle behaviours to manage metabolic syndrome. This scale consists of 36 items including physical activity, dietary behaviour, weight control, alcohol consumption and smoking, stress management and sleep and rest. Participants rate each item on a four‐point Likert‐type scale. The total score is calculated by averaging all item scores, with possible values ranging from 1 to 4. Higher scores indicate better lifestyle modification behaviours. The Cronbach's *α* was 0.92 in the original study (Kang 2010) and 0.93 in this study.

#### 3.3.3 Data Analysis

Data were analysed using SPSS for Windows (version 29) and AMOS software (version 26; IBM Corp., Armonk, NY, USA). Item analysis was first conducted with the total sample to generate the mean and standard deviation by item, skewness and kurtosis, item‐total correlation coefficients and Cronbach's *α* if item deleted. Items with low skewness and kurtosis (≤±1.97) (Yu 2012), strong item‐total correlation (≥ 0.30) and high Cronbach's *α* if item deleted (≥ 0.70) were retained (Hair et al. 2019; Nunnally Jr 1970).

EFA was performed to examine the underlying factor structure of the 24‐item IMI‐HeartLife. Prior to the analysis, the Kaiser–Meyer–Olkin (KMO) measure of sampling adequacy and Bartlett's test of sphericity confirmed the suitability of the data for factor analysis. The KMO value exceeded the acceptable threshold of 0.50 and Bartlett's test was significant (*p* < 0.05), indicating that the data were appropriate for factor analysis (Song 2015). A principal axis factoring extraction method was used, as it is appropriate for identifying latent constructs rather than maximising explained variance (Tavakol and Wetzel 2020). Given that the dimensions of the IMI‐HeartLife were theoretically expected to be correlated, an oblique rotation (Promax) was applied (DeVellis 2016). The number of factors to retain was determined based on multiple criteria, including eigenvalues greater than 1.0, scree plot inspection, parallel analysis and theoretical interpretability. In the parallel analysis, 1000 random datasets were generated with the same number of variables and participants, and eigenvalues from the actual data were compared with the mean eigenvalues from random data. Factors were retained if their actual eigenvalues exceeded the corresponding random eigenvalues. A cumulative variance explained exceeding 60% was considered acceptable (Hair et al. 2019). Items with factor loadings below 0.40, cross‐loadings (i.e., ≥ 0.32 of two factors with an absolute loading difference < 0.20), or communalities below 0.30 were considered for item removal sequentially while maintaining theoretical coherence (Hair et al. 2019).

CFA was conducted to evaluate the construct validity of the IMI‐HeartLife based on the factorial structure identified in the EFA. Model fit was assessed using multiple established indices, including the chi‐square statistic divided by degrees of freedom (CMIN/DF), standardised root mean square residual (SRMR), root mean square error of approximation (RMSEA), comparative fit index (CFI) and Tucker–Lewis index (TLI). Acceptable model fit was defined as CMIN/DF ≤ 5.0, SRMR ≤ 0.08, RMSEA ≤ 0.10, CFI and TLI ≥ 0.90 (Byrne 2012; Hu and Bentler 1999). During the model evaluation, a low squared multiple correlation (SMC < 0.40) (Jaschinski et al. 2021) was considered as the criterion for item removal to optimise the goodness‐of‐fit of the model and to improve convergent validity. This refinement can ensure that all retained items demonstrate adequate factor loadings and explained variance, contributing to a theoretically coherent and statistically robust model. The adequacy of individual items was evaluated using standardised factor loadings (*β* ≥ 0.32) and critical ratios (|CR| > 1.96, *p* < 0.05) (Hair et al. 2019). Construct reliability and average variance extracted (AVE) were computed to evaluate internal consistency and convergent validity, following the recommended cutoffs of 0.70 and 0.50 for AVE, respectively (Hair et al. 2019).

Discriminant validity was evaluated using the Korean version of the SIMS (Hwaing and Kim 2013). Pearson correlation analysis was conducted to test the hypothesis that IMI‐HeartLife scores would correlate most strongly with the intrinsic motivation subscale of the SIMS, followed by identified regulation, then external regulation, and would show a negative correlation with amotivation. Because the internal consistency coefficients (Cronbach's *α* = 0.56–0.94) of the SIMS subscales were relatively low in the present sample, correlations were corrected for attenuation to account for measurement error in both instruments. This adjustment allowed interpretation of associations independent of measurement unreliability. Furthermore, discriminant validity was assessed using both the Fornell–Larcker criterion and the heterotrait–monotrait (HTMT) ratio of correlations. According to the Fornell–Larcker criterion, the square root of the AVE for each factor was greater than its correlations with other factors, supporting discriminant validity (Fornell and Larcker 1981). In addition, all HTMT values were considered acceptable if below the conservative threshold of 0.85 (Henseler et al. 2015), indicating that the factors were empirically distinct.

Convergent validity was assessed using the Evaluation Tool for Metabolic Syndrome Modification Lifestyles (Kang 2010) by testing the hypothesis that higher levels of the IMI‐HeartLife would be significantly correlated with greater engagement in heart‐healthy lifestyle behaviours. Pearson correlation analysis was conducted to test the hypothesis.

Internal consistency was evaluated using Cronbach's *α* (≥ 0.70) (Hair et al. 2019), complemented by McDonald's *ω* coefficients and mean inter‐item correlations (MIC) for a more accurate estimation of reliability. The *ω*_total coefficient (criterion ≥ 0.70) was used to assess overall reliability (McDonald 1999), and the *ω*_hierarchical coefficient (criterion ≥ 0.50) was used to evaluate the proportion of reliable variance attributable to a general factor underlying the subscales (Zinbarg et al. 2006). This approach accounts for violations of tau‐equivalence and reduces potential overestimation from *α* values exceeding 0.90, which may indicate item redundancy. MIC values were examined against the recommended range of 0.15–0.50 (Boonmann et al. 2020). High *α* values accompanied by acceptable *ω* and MIC indices were interpreted as evidence of a well‐defined latent construct rather than redundancy. Test–retest reliability was assessed using the intraclass correlation coefficient (ICC [3,1]) based on a two‐way mixed‐effects model with single measurements to examine the consistency between the two administrations. A value of ≥ 0.70 was considered acceptable (Terwee et al. 2007). This model is appropriate when the same fixed measurement instrument is administered to the same participants at both time points (Koo and Li 2016). To evaluate measurement precision, the standard error of measurement (SEM) and minimal detectable change (MDC) were computed. The SEM was calculated using the formula SEM = SD × √(1 – ICC), where SD is the standard deviation of the total score (van Kampen et al. 2013). The MDC at the 95% confidence level was estimated as MDC_95_ = 1.96 × √2 × SEM, which represents the smallest detectable change beyond measurement error (van Kampen et al. 2013).

Measurement invariance of the IMI‐HeartLife was evaluated across gender using multi‐group CFA with the total sample (*N* = 703; men = 359, women = 344). The analysis followed a hierarchical sequence of models—configural, metric, and scalar invariance—to determine whether the factor structure, factor loadings and item intercepts were equivalent across gender groups. First, configural invariance (Model 1) tested whether the same factorial structure (number of factors and pattern of item loadings) was applicable across male and female groups without constraining any parameters. Next, metric invariance (Model 2) constrained factor loadings to be equal across groups, and scalar invariance (Model 3) further constrained both factor loadings and intercepts. Model fit was evaluated using the CFI, RMSEA and SRMR. Measurement invariance was considered supported when the change in model fit indices between nested models met the recommended criteria: ΔCFI ≤ 0.010, ΔRMSEA ≤ 0.015 and ΔSRMR ≤ 0.030 for metric invariance and ≤ 0.010 for scalar invariance (Chen 2007).

Prior to analysis, data quality was verified. Because the survey was conducted through a professional online survey agency, only participants who completed all questionnaire items were able to submit the questionnaire. Therefore, there were no missing responses in the dataset, and complete‐case analysis was applied throughout.

### 3.4 Ethical Consideration

This study was approved by the Institutional Review Board of Korea University (No. KUIRB‐2022‐0418‐01), and all procedures were conducted in accordance with the ethical standards set by the board. Written informed consent was obtained from all participants prior to their involvement, and they received remuneration for their participation.

## 4 Results

### 4.1 General Characteristics of the Participants

Table 1 presents the general characteristics of the total sample (*N* = 703), including Subsample 1 for EFA and Subsample 2 for CFA. There were no significant differences between the two subsamples on any of the reported variables.

**TABLE 1:** General characteristics of the participants (*N* = 703).

	*n* (%) or mean (SD)			*χ* ^2^ or *t*	*p*
	Total	Subsample 1 (*n* = 352)	Subsample 2 (*n* = 351)		
Age (years)	42.7 (12.96)	42.9 (12.72)	42.5 (13.22)	0.46	0.643
Gender					
Men	359 (51.1)	175 (49.7)	184 (52.4)	0.52	0.473
Women	344 (48.9)	177 (50.3)	167 (47.6)		
Education					
< College educated	394 (56.0)	196 (55.7)	198 (56.4)	0.04	0.846
≥ College educated	309 (44.0)	156 (44.3)	153 (43.6)		
Monthly household income					
≤ 5 million KRW	387 (55.0)	201 (57.1)	186 (53.0)	1.20	0.273
> 5 million KRW	316 (45.0)	151 (42.9)	165 (47.0)		
Employed status					
Yes	532 (75.7)	275 (78.1)	257 (73.2)	2.30	0.130
No	171 (24.3)	77 (21.9)	94 (26.8)		
Underlying diseases					
HTN, DM, hyperlipidaemia	133 (18.9)	62 (17.6)	71 (20.2)	0.87	0.646
Others	158 (22.5)	82 (23.3)	76 (21.7)		
None	412 (58.6)	208 (59.1)	204 (58.1)		

*Note:* KRW refers to the currency unit of South Korea.

Abbreviations: DM, diabetes mellitus; HTN, hypertension; KRW, Korean won; SD, standard deviation.

### 4.2 Content and Face Validity

In the first round of expert evaluations, 14 of the 32 items in the initial pool of items did not meet the I‐CVI threshold of 0.80 in terms of either relevance or clarity. Rather than eliminating these items, the authors chose to proceed to a second round of expert review after 26 out of the 32 items were reworded or rephrased to improve clarity and relevance. In the second round, the I‐CVI for relevance ranged from 0.88 to 1.00, whereas the S‐CVI for relevance was 0.97. The I‐CVI for clarity ranged from 0.75 to 1.00, whereas the S‐CVI for clarity was 0.91. Based on the I‐CVI for clarity, four items (Items 18, 21, 31, and 32) were removed due to redundancy and interpretational difficulty arising from reverse wording or ambiguous phrasing (Tables S1 and S3). Next, 28 items were assessed in a pilot test. Seven items (Items 4, 8, 12, 15, 16, 22, & 24) were refined to eliminate redundancy and improve consistency in wording. All 28 items were reviewed by a Korean linguist to ensure vocabulary accuracy and appropriateness of expression, and two items (Item 15 & 21) were further refined to enhance linguistic accuracy and fluency. All items were finalised through consensus by three researchers. As a result, a total of 28 consolidated items were selected for the next phase.

### 4.3 Psychometric Evaluation: Construct Validity

Among the 28 items, none met the threshold for deletion based on distributional criteria (Yu 2012) (Table 2). Four items (Items 4, 11, 23, & 28) were removed because their item–total correlations fell below the acceptable threshold of ≥ 0.30 (Hair et al. 2019) (Tables S1 and S3). The Cronbach's *α* values if each item was deleted ranged from 0.91 to 0.93, indicating that removal of any single item did not improve the overall internal consistency. Consequently, a total of 24 items retained.

**TABLE 2:** Item analysis of the IMI‐HeartLife scale using 28 items (*N* = 703).

Items	Mean ± SD	Skewness	Kurtosis	Correlation coefficient between item and total score (*r*)	Reliability when item deleted (Cronbach's *α*)
01	4.4 ± 1.41	−0.25	−0.43	0.73**	0.91
02	5.5 ± 1.17	−0.76	0.41	0.55**	0.92
03	4.4 ± 1.35	−0.10	−0.33	0.73**	0.92
04	3.9 ± 1.40	0.04	−0.51	−0.29**	0.93
05	4.1 ± 1.53	−0.19	−0.64	0.73**	0.91
06	4.1 ± 1.43	−0.01	−0.44	0.72**	0.92
07	5.6 ± 1.14	−0.86	0.88	0.51**	0.92
08	4.8 ± 1.51	−0.51	−0.25	0.80**	0.91
09	5.1 ± 1.39	−0.70	0.22	0.72**	0.92
10	5.3 ± 1.37	−0.69	0.21	0.78**	0.91
11	3.2 ± 1.44	0.43	−0.30	−0.35**	0.93
12	3.7 ± 1.57	0.14	−0.74	0.37**	0.92
13	4.2 ± 1.54	−0.19	−0.63	0.74**	0.91
14	5.5 ± 1.19	−0.74	0.56	0.70**	0.92
15	5.5 ± 1.18	−0.69	0.40	0.59**	0.92
16	4.2 ± 1.52	−0.06	−0.55	0.68**	0.92
17	5.7 ± 1.17	−0.81	0.41	0.58**	0.92
19	4.6 ± 1.41	−0.37	−0.20	0.75**	0.91
20	4.6 ± 1.49	−0.43	−0.44	0.78**	0.91
22	4.6 ± 1.32	−0.40	0.15	0.64**	0.92
23	3.4 ± 1.50	0.31	−0.37	−0.45**	0.93
24	4.2 ± 1.44	−0.01	−0.40	0.73**	0.91
25	4.8 ± 1.30	−0.43	0.36	0.71**	0.92
26	5.5 ± 1.29	−0.85	0.78	0.59**	0.92
27	4.3 ± 1.54	−0.16	−0.67	0.77**	0.91
28	4.4 ± 1.54	−0.19	−0.69	0.04**	0.93
29	4.5 ± 1.59	−0.27	−0.65	0.50**	0.92
30	5.6 ± 1.18	−0.80	0.59	0.58**	0.92

Abbreviation: SD, standard deviation.

** *p* < 0.001.

**FIGURE 2:**
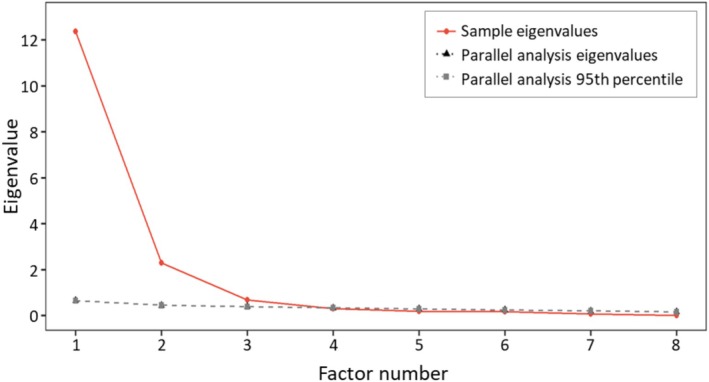
Scree plot and parallel analysis based on eigenvalues with Subsample 1 (*n* = 352).

The 24 items were evaluated using EFA (Table 3). The KMO measure of sampling adequacy was 0.95, exceeding the acceptable threshold of 0.50 and Bartlett's test of sphericity was significant (*χ* ^2^ = 7630.30, *p* < 0.001), confirming the suitability of the data for factor analysis (Song 2015). Using principal axis factoring with Promax rotation, three factors were extracted, generally consistent with the theoretical structure of the IMI‐HeartLife. The eigenvalues of the three retained factors were greater than 1.0 (Table 3), and both the scree plot (Figure 2) and parallel analysis supported a three‐factor solution. The scree plot displayed a clear inflection after the third factor, and the parallel analysis indicated a three‐factor solution, as the first three eigenvalues from the actual data (12.36, 2.30 and 0.69) exceeded the corresponding randomly generated eigenvalues (0.65, 0.44 and 0.38, respectively), supporting the retention of three factors. During the EFA process, five items were removed based on psychometric criteria: one item (Item 22) due to a low factor loading (< 0.40), three items (Items 9, 10 and 27) due to cross‐loadings, and one item for low communality (Tables S1 and S3). The final EFA solution comprised 19 items grouped into three factors (Table 3): Factor 1—Interest/Enjoyment (5 items), Factor 2—Choice/Value/Usefulness (7 items) and Factor 3—Competence/Effort (4 items). The three factors accounted for 70.4% of the total variance, exceeding the recommended 60% threshold for construct validity (Hair et al. 2019). Inter‐factor correlations ranged from 0.88 to 0.91, indicating that the subscales were highly correlated yet theoretically distinct dimensions of the IMI‐HeartLife.

**TABLE 3:** Exploratory factor analysis with 24 items using Subsample 1 (*n* = 352).

Items	Factor loading			Communality
	Factor 1	Factor 2	Factor 3	
3	−0.62			0.69
6	−0.86			0.77
16	−0.96			0.76
19	−0.61			0.70
24	−0.80			0.78
2		0.67		0.54
7		0.83		0.63
10^a^		0.50	0.40	0.71
14		0.65		0.68
15		0.66		0.57
17		0.92		0.76
26		0.81		0.70
30		0.92		0.79
1			0.73	0.70
5	−0.37		0.58	0.71
8			0.58	0.70
9^a^		0.38	0.52	0.62
13	−0.34		0.61	0.74
20			0.71	0.79
22^b^			0.39	0.57
25			0.44	0.63
27^a^	−0.40		0.52	0.76
29			0.68	0.39
12^c^			0.44	0.19
Eigenvalue	1.19	2.90	12.80	
% of total variance	5.0	12.1	53.3	
Cumulative percentages	5.0	17.1	70.4	
Bartlett's test of sphericity	*χ* ^2^ = 7630.30, *p* < 0.001			
KMO measure	0.95			
Factor—total correlation coefficient	0.88 (< 0.001)	0.78 (< 0.001)	0.91 (< 0.001)	

*Note:* EFA, conducted suing principal axis factoring and Promax. Items marked ^a,b^ or ^c^ were removed.

^a^ Items with cross‐loadings ≥ 0.32 on two factors with loading differences ≤ 0.20.

^b^ Items with all factor loadings < 0.40 across factors.

^c^ Items with communality < 0.30; KMO = Kaiser–Meyer–Olkin measure of sampling adequacy.

**FIGURE 3:**
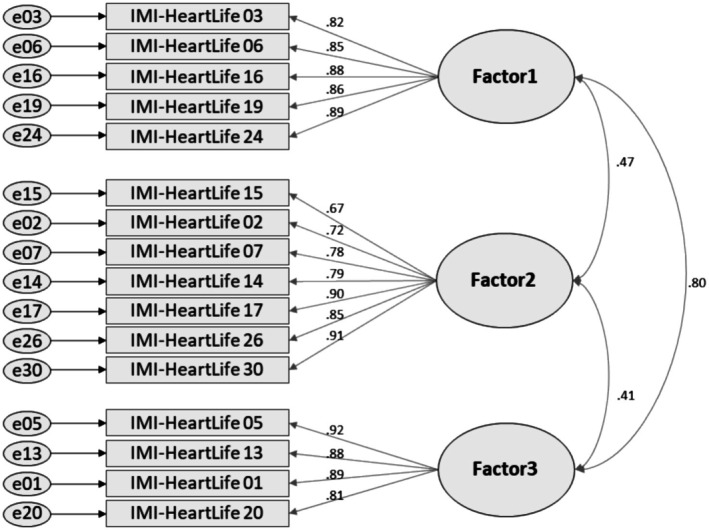
Confirmatory factor analysis for the IMI‐HeartLife using Subsample 2 (*n* = 351). IMI‐HeartLife, Intrinsic Motivation Inventory for Heart‐Healthy Lifestyle. Factor 1 = Interest/Enjoyment; Factor 2 = Perceived Choice/Value/Usefulness; Factor 3 = Perceived Competence/Effort.

The 19 items identified through EFA were further evaluated using CFA (Table 4). The initial model showed suboptimal fit (CMIN/DF = 4.87, SRMR = 0.12, RMSEA = 0.11, CFI = 0.90, TLI = 0.88). Three items (Items 8, 25 and 29) with low squared multiple correlations (SMC < 0.40) were removed (Hair et al. 2019; Tables S1 and S3), resulting in a final model with 16 retained items. The revised 16‐item model (Figure 3) demonstrated good model fit (CMIN/DF = 3.43, SRMR = 0.06, RMSEA = 0.08, CFI = 0.95, TLI = 0.94; Table 4). All standardised factor loadings exceeded 0.32 (range = 0.67–0.92), and all critical ratios were greater than ±1.96, indicating statistical significance. Each factor showed high construct reliability (0.93, exceeding the 0.70 criterion) and AVE (0.65–0.74. exceeding the 0.50 criterion), supporting excellent internal consistency across all constructs (Table 4).

**TABLE 4:** Confirmatory factor analysis using 16 items with Subsample 2 (*n* = 351).

Factors	Items		*β*	Critical ratio	Factors *r* (HTMT)			√AVE	AVE	Construct reliability
					1	2	3			
1	Interest/Enjoyment							0.86	0.74	0.93
	3	I really enjoyed practicing a healthy lifestyle.	0.82	—						
	6	Practicing a healthy lifestyle was fun.	0.85	19.19						
	16	I would say that practicing a healthy lifestyle was very interesting.	0.88	20.47						
	19	I thought practicing a healthy lifestyle was enjoyable.	0.86	19.52						
	24	While practicing a healthy lifestyle, I felt it was very enjoyable.	0.89	20.83						
2	Choice/Value/Usefulness				0.47* (0.53)			0.80	0.65	0.93
	15	I believe I have a choice in practicing a healthy lifestyle.	0.67	—						
	2	I think practicing a healthy lifestyle is an important activity for my heart health.	0.72	12.35						
	7	I think practicing a healthy lifestyle is useful for my heart health.	0.78	13.24						
	14	I believe practicing a healthy lifestyle is valuable to me.	0.79	13.39						
	17	I think practicing a healthy lifestyle could help improve my heart health.	0.90	14.91						
	26	I think it's important to practice a healthy lifestyle because it helps with heart health.	0.85	14.22						
	30	I believe practicing a healthy lifestyle could be beneficial for my heart health.	0.91	15.04						
3	Competence/Effort				0.80* (0.83)	0.41* (0.49)		0.82	0.68	0.93
	5	I think I'm good at practicing a healthy lifestyle.	0.92	—						
	13	I think I practice a healthy lifestyle well compared to others.	0.88	25.69						
	1	I put a lot of effort into practicing a healthy lifestyle.	0.89	25.98						
	20	I tried very hard to practice a healthy lifestyle.	0.81	21.37						
Goodness‐of‐fit		CMIN/DF = 3.43, SRMR = 0.06, RMSEA = 0.08, CFI = 0.95, TLI = 0.94								

Abbreviations: AVE, average variance extracted; CFI, comparative fit index; CMIN/DF = normed *χ* ^2^; HTMT, Heterotrait‐monotrait ratio of correlations; RMSEA, root mean squared error of approximation; SRMR, standardised root mean squared residual; *β*, standardised factor loading.

* *p* < 0.001.

Discriminant validity was supported through multiple approaches. First, Pearson correlation analyses using the Korean version of the SIMS (Hwaing and Kim 2013) showed that IMI‐HeartLife total and subscale scores correlated most strongly with the intrinsic motivation subscale of the SIMS (*r* = 0.0.56, *p* < 0.001), followed by identified regulation (*r* = 0.55, *p* < 0.001), and less strongly with external regulation (*r* = 0.09, *p* = 0.017). Correlations with amotivation were negative as hypothesised (*r* = −0.27, *p* < 0.001) (Table 5). After correcting for attenuation to account for measurement error in both instruments, the correlation pattern showed a slight change (Table 5). Specifically, the IMI‐HeartLife scores correlated most strongly with the identified regulation subscale of the SIMS (*r* = 0.76, *p* < 0.001), followed by intrinsic motivation (*r* = 0.60, *p* < 0.001), indicating a reversal in the expected order of associations. Second, discriminant validity among the IMI‐HeartLife factors was further examined using the Fornell–Larcker and HTMT criteria (Table 4). The square root of the AVE for each factor exceeded its correlations with the other factors, satisfying the Fornell–Larcker criterion (Fornell and Larcker 1981). All HTMT ratios of correlations were below 0.85 (ranging from 0.49 to 0.83), indicating that the three subscales—Interest/Enjoyment, Choice/Value/Usefulness and Competence/Effort—were empirically distinct yet conceptually related dimensions of intrinsic motivation (Henseler et al. 2015).

**TABLE 5:** Discriminant and convergent validity of the IMI‐HeartLife: Pearson's correlation coefficients between scales (*N* = 703).

Measures		*r* (*p*)	*r* * (*p*)
Discriminant validity	Situational Motivation Scale, overall	0.50 (< 0.001)	0.56 (< 0.001)
	Intrinsic motivation	0.56 (< 0.001)	0.60 (< 0.001)
	Identified regulation	0.55 (< 0.001)	0.76 (< 0.001)
	External regulation	0.09 (0.017)	0.12 (0.017)
	Amotivation	−0.27 (< 0.001)	−0.31 (< 0.001)
Convergent validity	Evaluation tool for metabolic syndrome modification lifestyles	0.69 (< 0.001)	—

Abbreviations: IMI‐HeartLife, Intrinsic Motivation Inventory for Heart‐Healthy Lifestyle; SD, standard deviation.

* Correct correlations for attenuation.

Convergent validity revealed that IMI‐HeartLife was significantly correlated with the total score of the Evaluation Tool for Metabolic Syndrome Modification Lifestyles (*r* = 0.69, *p* < 0.001) (Table 5). The convergent validity of the scale can be considered acceptable.

### 4.4 Psychometric Evaluation: Internal Consistency and Test–Retest Reliability

Internal consistency of the IMI‐HeartLife was excellent across the overall scale and subscales (Table 6). Cronbach's *α* values were 0.94 for the total scale and 0.93 for the subscales, exceeding the minimum criterion of 0.70 (Hair et al. 2019). Although *α* values greater than 0.90 may raise concerns of potential item redundancy (Hair et al. 2019), complementary indices provided further evidence of structural coherence. The *ω*_total values (0.94–0.97) indicated excellent composite reliability, reflecting the proportion of variance explained by all common factors, whereas the *ω*_hierarchical value of the total IMI‐HeartLife scale (0.74), which exceeded the recommended threshold of 0.50, demonstrated that a substantial portion of reliable variance was attributable to a general factor rather than item overlap (McDonald 1999; Zinbarg et al. 2006). Moreover, the MIC of the overall IMI‐HeartLife (0.50) fell within the recommended range of 0.15–0.50 (Boonmann et al. 2020), supporting adequate internal consistency without excessive homogeneity. Collectively, these findings indicate that the IMI‐HeartLife exhibits a well‐defined latent construct and strong internal coherence, rather than redundancy among items.

**TABLE 6:** Reliability of the IMI‐HeartLife: Internal consistency and test–retest reliability.

Factors	Internal consistency (*N* = 703)				Test–retest reliability (*n* = 50)		
	Cronbach's *α*	*ω*_total	*ω*_hierarchical	Mean inter‐item correlation	ICC (3,1) (95% CI)	SEM	MDC_95_
IMI‐HeartLife, total	0.94	0.97	0.74	0.50	0.84 (0.73–0.91)	0.36	0.99
FACTOR 1: interest/enjoyment	0.93	0.94	0.94	0.73	0.72 (0.55–0.83)	0.58	1.60
FACTOR 2: choice/value/usefulness	0.93	0.94	0.94	0.65	0.82 (0.70–0.89)	0.39	1.08
FACTOR 3: competence/effort	0.93	0.94	0.94	0.76	0.83 (0.72–0.90)	0.51	1.41

Abbreviations: CI, confidence interval; ICC, intraclass correlation coefficient; IMI‐HeartLife, Intrinsic Motivation Inventory for Heart‐Healthy Lifestyle; MDC_95_, minimal detectable change at the 95% confidence level; SEM, standard error of measurement.

The test–retest reliability of the IMI‐HeartLife was satisfactory (Table 6), with ICC (3,1) values of 0.84 for the total scale and ranging from 0.72 to 0.83 across the subscales, all exceeding the acceptable criterion of 0.70 (Terwee et al. 2007). These results indicate good temporal stability of the instrument when administered twice under equivalent conditions (Koo and Li 2016). Regarding measurement precision, the SEM was 0.36 for the total IMI‐HeartLife and ranged from 0.39 to 0.58 across the subscales, suggesting a low level of measurement error. The corresponding MDC values at the 95% confidence level were 0.99 for the total scale and between 1.08 and 1.60 for the subscales, indicating that score changes exceeding these values can be interpreted as true changes rather than random variation or measurement error (van Kampen et al. 2013).

### 4.5 Multi‐Group CFA for Measurement Invariance Evaluation Across Gender

The results of the multi‐group CFA supported measurement invariance of the IMI‐HeartLife across gender (Table S4). The configural invariance model (M1) demonstrated acceptable model fit (CFI = 0.931, RMSEA = 0.069, SRMR = 0.063), indicating that the factorial structure was equivalent across men and women. When factor loadings were constrained to equality (metric invariance, M2), model fit remained largely unchanged (ΔCFI = 0.001, ΔRMSEA = 0.001, ΔSRMR = 0.003). Further constraining item intercepts (scalar invariance, M3) also produced negligible differences in fit indices (ΔCFI = 0.000, ΔRMSEA = 0.002, ΔSRMR = 0.000). All changes met the established cut‐off criteria, providing strong evidence that the IMI‐HeartLife demonstrates configural, metric and scalar invariance across gender, and thus measures the same latent constructs equivalently among men and women.

## 5 Discussion

The IMI‐HeartLife demonstrated a three‐factor structure comprising 16 items measured on a seven‐point Likert scale. Its psychometric properties indicated good construct validity and reliability. The full English and Korean versions of the IMI‐HeartLife, along with the scoring instructions, are presented in the Supporting Information Appendix to enhance transparency and facilitate replication in future research.

A coherent three‐factor structure of the IMI‐HeartLife—Interest/Enjoyment, Choice/Value/Usefulness and Competence/Effort—was confirmed, aligning with the theoretical foundations of the original IMI with the Self‐Determination Theory, and demonstrating good model fit. Among the seven original IMI subscales, five were selected and conceptually integrated into three broader factors relevant to the context of heart‐healthy lifestyle behaviours: (1) Interest/Enjoyment, (2) the integration of Perceived Choice and Value/Usefulness and (3) the integration of Perceived Competence and Effort/Importance. Meanwhile, the exclusion of the Pressure/Tension and Relatedness subscales from the original IMI during the conceptualisation phase may weaken theoretical coherence to some extent. Nevertheless, this decision can be justified by the conceptual specificity of intrinsic motivation and the self‐directed nature of heart‐healthy lifestyle behaviours. The excluded subscales represent constructs theoretically distinct from intrinsic motivation along the self‐regulation continuum: Pressure/Tension reflects controlled or introjected regulation, whereas Relatedness captures interpersonal connectedness rather than autonomous engagement. However, the Relatedness dimension may still hold theoretical relevance, as social support and reinforcement from significant others—such as family members, peers and colleagues—have been shown to enhance motivation for initiating and maintaining health‐promoting behaviours (Collazo‐Castiñeira et al. 2025; Worthen‐Chaudhari et al. 2024). If incorporated into the IMI‐HeartLife, relatedness would conceptually function as a contextual facilitator of identified or integrated regulation, rather than as a direct component of intrinsic motivation. Indeed, social support is closely associated with health behaviours, and recent systematic reviews (Babygeetha and Devineni 2024) have highlighted that, among patients with coronary artery disease and heart failure, support from family members and access to psychological resources are key facilitators of self‐care behaviours. From this perspective, future iterations of the IMI‐HeartLife should consider reintegrating the Relatedness subscale with newly developed items and re‐evaluating its contribution to the overall factor structure.

Regarding the Choice/Value/Usefulness subscale, its conceptual proximity to identified regulation rather than pure intrinsic motivation may be acknowledged. In the context of adopting and maintaining heart‐healthy behaviours, this factor represents an autonomous form of motivation characterised by volitional choice and personally endorsed health values. This interpretation aligns with Self‐Determination Theory's broader continuum, wherein intrinsic and identified regulations jointly reflect autonomous motivation. Accordingly, motivation for heart‐healthy lifestyle engagement may be best understood as an identified‐autonomous orientation, integrating both intrinsic enjoyment and value‐based volition. This perspective supports the theoretical coherence of the IMI‐HeartLife in capturing the multifaceted nature of autonomous motivation underlying sustained health behaviour change.

The discriminant validity of the IMI‐HeartLife was acceptable. The hypothesised discriminant validity pattern suggested that the IMI‐HeartLife would show the highest correlation with the intrinsic motivation subscale of the SIMS; however, after correction for attenuation, the strongest correlation was instead observed with identified regulation, while the expected discriminant relationships with the other SIMS subscales were maintained. This slight reversal can be interpreted within the theoretical continuum of Self‐Determination Theory. Identified regulation represents a relatively autonomous form of extrinsic motivation, situated on the autonomous end of the self‐regulation continuum, and is characterised by personal endorsement and valuing of the behaviour (Deci and Ryan 2000). In the context of heart‐healthy lifestyle behaviours, individuals may engage in such activities not merely for intrinsic enjoyment but also out of an internalised recognition of their significance to health and overall well‐being. Therefore, the stronger corrected correlation with identified regulation likely reflects the goal‐directed and health‐related nature of the IMI‐HeartLife construct rather than a psychometric overlap or lack of discriminant validity. Consistent with previous research showing that intrinsic and identified regulations often covary in health‐behaviour domains where actions are instrumental yet self‐endorsed (Teixeira et al. 2012), this pattern suggests that while the IMI‐HeartLife primarily captures intrinsic motivation, it also aligns closely with internalised health values and self‐determined reasons for practicing heart‐healthy behaviours—supporting the theoretical coherence of the scale within SDT.

The convergent validity was found to be strong, as evidenced by a high correlation (*r* = 0.69) of the IMI‐HeartLife with the comprehensive heart‐healthy behaviours. While few prior studies have examined the relation between motivation and composite health behaviours, some evidence—such as a systematic review indicating that motivational traits like conscientiousness are associated with increased physical activity—supports this association (Macali et al. 2025). Finaly, our finding suggests that intrinsic motivation may play a pivotal role in initiating and sustaining comprehensive heart‐healthy lifestyle behaviours.

Reliability evidence for the IMI‐HeartLife was robust, reflecting both internal consistency and test–retest reliability (i.e., temporal stability). The scale demonstrated strong coherence across subscales, with internal consistency indices exceeding recommended thresholds for Cronbach's *α* and McDonald's *ω*, and mean inter‐item correlations indicating appropriate homogeneity without redundancy. Furthermore, the test–retest analysis yielded high intraclass correlation coefficients, confirming the instrument's stability over time. Collectively, these findings suggest that the IMI‐HeartLife is a psychometrically sound and stable measure of intrinsic motivation for heart‐healthy lifestyle behaviours, supporting its applicability in both research and practice.

We had study limitations. First, concerns may be raised regarding the generalisability of the instrument. Methodologically, the study recruited participants from the general population without diagnosed cardiovascular disease, providing a reasonable degree of representativeness for the Korean adult population. However, further validation is warranted to examine measurement invariance across additional demographic groups such as age (gender invariance was tested in the present study) and to confirm cross‐cultural applicability beyond South Korea. Second, as the data were collected via an online survey, the instrument may be particularly suited for digital administration. Nonetheless, responses may vary with paper‐based administration, underscoring the need for future studies to revalidate the scale using alternative delivery formats. In addition, although the total sample can be regarded as broadly representative of the Korean adult population, it was limited to individuals with access to online platforms. Therefore, potential digital access inequalities should be considered when interpreting the findings, as such disparities may influence participation and response patterns. Third, the IMI‐HeartLife did not include items representing the Relatedness or Pressure/Tension subscales from the original IMI. This decision was based on contextual considerations and the theoretical inappropriateness of these constructs for assessing intrinsic motivation underlying autonomous regulation of heart‐healthy lifestyle behaviours, as detailed in the Methods section. Future research may develop context‐specific items to capture these dimensions when expanding the IMI‐HeartLife across the broader self‐determination continuum. Fourth, the Korean version of the Situational Motivation Scale used as a comparator demonstrated relatively low internal consistency for some subscales (Cronbach's *α* as low as 0.56), which may have attenuated the observed correlations and partially affected the conclusions regarding discriminant validity. Although we statistically corrected for attenuation to account for measurement error, this adjustment cannot fully compensate for the unreliability of the external instrument. Therefore, the interpretation of discriminant validity should be made with caution, and future studies are encouraged to employ comparator instruments with well‐established reliability to confirm the robustness of these findings.

## 6 Conclusion

The IMI‐HeartLife is a psychometrically sound instrument for assessing intrinsic motivation to promote heart‐healthy lifestyle behaviours aimed at preventing cardiovascular disease, grounded in the theoretical framework of Self‐Determination Theory. It consists of three subscales: Interest/Enjoyment, Choice/Value/Usefulness and Competence/Effort. Future research may aim to refine the scale to improve its comprehensiveness and examine its applicability across a wider range of health promotion contexts, including motivation‐enhancing interventions.

The IMI‐HeartLife offers nurses a practical and validated instrument to assess the intrinsic motivation of individuals engaging in heart‐healthy behaviours. By integrating this tool into clinical assessments, nurses can shift from being prescriptive information providers to collaborative facilitators of motivation. Through this process, nurses can utilise the tool as a guide to identify individuals' motivational levels and types, and to deliver personalised behavioural interventions aimed at enhancing motivation—particularly among high‐risk individuals in the community without cardiovascular disease, as well as patients preparing for discharge or participating in outpatient‐based cardiac rehabilitation programs, where sustained behaviour modification is essential for cardiovascular disease prevention.

## Author Contributions

**Jina Choo:** conceptualisation, writing – original draft, writing – review and editing, validation, project administration, supervision, methodology and funding acquisition. **Songwhi Noh:** writing – review and editing, project administration, data curation, resources and formal analysis. **Yura Shin:** writing – review and editing, software, visualisation and formal analysis.

## Funding

This work was supported by National Research Foundation of Korea (NRF) grant from the Korean Government (NRF‐2019R1A2C1004116) and National Research Foundation of Korea (NRF) grant from the Ministry of Science and ICT (RS‐2024‐00336847).

## Disclosure

Statement on number of references: Over 25 references were cited to situate the adaptation and validation of the IMI‐HeartLife within Self‐Determination Theory, cardiovascular health interventions and psychometric frameworks and to substantiate the interpretation of results across behavioural and health promotion domains.

There is a statistician on the author team and state which author: A statistician is included in the author team. Dr. Jina Choo (PhD, DrPH), an epidemiologist and statistical expert with additional expertise in psychometric methodology for instrument development, reviewed and validated all statistical analyses to ensure accuracy and methodological rigour. Furthermore, expert consultation from a psychometric statistician was obtained throughout the development and validation process to enhance the analytical robustness of the study.

## Consent

Written informed consent was obtained from all participants prior to data collection, and they received remuneration for their participation.

## Conflicts of Interest

The authors declare no conflicts of interest.

## Supporting information


**Data S1:** nop270406‐sup‐0001‐DataS1.docx.

## Data Availability

The data that support the findings of this study are available from the corresponding author upon reasonable request.
