# Supplementary material for: Intrinsic Motivation Inventory for Heart‐Healthy Lifestyle (IMI‐HeartLife) in Cardiovascular Disease Prevention: A Validation Study
Source: Nurs Open. 2025 Dec 23;12(12):e70406. doi: 10.1002/nop2.70406 (PMC12723749; doi:10.1002/nop2.70406)
Supplement: Supplementary file 1 — Data S1: nop270406‐sup‐0001‐DataS1.docx. [file NOP2-12-e70406-s001.docx]

| Supplementary Table 1. An initial pool of 32 items for the IMI-HeartLife | | | |
| --- | --- | --- | --- |
| Items no. in phase 1 | When deleted | Item statements | Reverse scored |
| 1 |  | I put a lot of effort into practicing a healthy lifestyle. |  |
| 2 |  | I think practicing a healthy lifestyle is an important activity for my heart health. | |
| 3 |  | I really enjoyed practicing a healthy lifestyle. |  |
| 4 | Phase 3 | I didn’t put much energy into practicing a healthy lifestyle. | (R) |
| 5 |  | I think I’m good at practicing a healthy lifestyle. |  |
| 6 |  | Practicing a healthy lifestyle was fun. |  |
| 7 |  | I think practicing a healthy lifestyle is useful for my heart health. |  |
| 8 | Phase 3 | I practiced a healthy lifestyle because I wanted to. |  |
| 9 | Phase 3 | It was important for me to practice a healthy lifestyle well. |  |
| 10 | Phase 3 | I would be willing to practice a healthy lifestyle again because it’s valuable to me. | |
| 11 | Phase 3 | I felt I had to practice a healthy lifestyle, regardless of my own will. | (R) |
| 12 | Phase 3 | Practicing a healthy lifestyle was not something I was good at. | (R) |
| 13 |  | I think I practice a healthy lifestyle well compared to others. |  |
| 14 |  | I believe practicing a healthy lifestyle is valuable to me. |  |
| 15 |  | I believe I have a choice in practicing a healthy lifestyle. |  |
| 16 |  | I would say that practicing a healthy lifestyle was very interesting. |  |
| 17 |  | I think practicing a healthy lifestyle could help improve my heart health. |  |
| 18 | Phase 2 | Practicing a healthy lifestyle did not interest me at all. | (R) |
| 19 |  | I thought practicing a healthy lifestyle was enjoyable. |  |
| 20 |  | I tried very hard to practice a healthy lifestyle. |  |
| 21 | Phase 2 | I did not really have a choice in practicing a healthy lifestyle. | (R) |
| 22 | Phase 3 | After practicing a healthy lifestyle for a while, I felt quite competent. |  |
| 23 | Phase 3 | I practiced a healthy lifestyle because I had to. | (R) |
| 24 |  | While practicing a healthy lifestyle, I felt it was very enjoyable. |  |
| 25 | Phase 3 | I’m satisfied with my performance in practicing a healthy lifestyle. |  |
| 26 |  | I think it’s important to practice a healthy lifestyle because it helps with heart health. | |
| 27 | Phase 3 | I am skilled at practicing a healthy lifestyle. |  |
| 28 | Phase 3 | I practiced a healthy lifestyle not by choice, but because I had no other option. | (R) |
| 29 | Phase 3 | I did not try very hard to practice a healthy lifestyle. | (R) |
| 30 |  | I believe practicing a healthy lifestyle could be beneficial for my heart health. | |
| 31 | Phase 2 | I thought practicing a healthy lifestyle was boring. | (R) |
| 32 | Phase 2 | I felt that practicing a healthy lifestyle was unrelated to my own choice. | (R) |
| Note: Items highlighted in the table indicate those that were retained in the finalized version of the IMI-HeartLife.  IMI-HeartLife = Intrinsic Motivation Inventory for Heart-Healthy Lifestyle  The study procedure according to established scale development guidelines in three phases: Phase 1=conceptualization and item pool generation; Phase 2= evaluation of the content validity and face validity; Phase 3 = psychometric evaluation. | | | |

| Supplementary Table 2. Representativeness of the Subsample (n = 50) Compared with the Total Sample (N = 703) for the Evaluation of Test–Retest Reliability | | | | |
| --- | --- | --- | --- | --- |
|  | n (%) or Mean (SD) | | χ^2^ or t | p |
|  | Total  (n = 703) | Subsample  (n = 50) |  |  |
| Age (years) | 42.7 (12.96) | 43.2 (12.4) | 0.24 | .810 |
| Gender |  |  |  |  |
| Men | 359 (51.1) | 25 (50.0) | 0.02 | .884 |
| Women | 344 (48.9) | 25 (50.0) |  |  |
| Education |  |  |  |  |
| < College educated | 394 (56.0) | 28 (56.0) | 0.00 | .995 |
| ≥ College educated | 309 (44.0) | 22 (44.0) |  |  |
| Monthly household income |  |  |  |  |
| < 5 million KRW | 387 (55.0) | 24 (48.0) | 0.67 | .412 |
| > 5 million KRW | 316 (45.0) | 26 (52.0) |  |  |
| Employed status |  |  |  |  |
| Yes | 532 (75.7) | 39 (78.0) | 0.14 | .711 |
| No | 171 (24.3) | 11 (22.0) |  |  |
| Underlying diseases |  |  |  |  |
| HTN, DM, hyperlipidaemia | 133 (18.9) | 6 (12.0) | 2.65 | .265 |
| Others | 158 (22.5) | 9 (18.0) |  |  |
| None | 412 (58.6) | 35 (70.0) |  |  |
| SD = standard deviation; DM = Diabetes mellitus; HTN = Hypertension; KRW = Korean won; KRW refers to the currency unit of South Korea. | | | | |

| Supplementary Table 3. Summary of the item reduction process for the IMI-HeartLife | | | | |
| --- | --- | --- | --- | --- |
| Initial items (n) | Step or analysis | Items removed (n)  (item no.) | Criterion for item reduction | Items retained (n) |
| 32 | Content validity | 4 (18, 21, 31, & 32) | I-CVI for clarity < 0.80 | 28 |
| 28 | Item analysis | 4 (4, 11, 23, & 28) | Item-total correlation < 0.30 | 24 |
| 24 | EFA | 1 (22) | Factor loading < 0.40 across factors |  |
|  |  | 3 (9, 10, & 27) | cross-loadings ≥ 0.32 on two factors with loading differences ≤ 0.20 |  |
|  |  | 1 (12) | Communality < 0.30 | 19 |
| 19 | CFA | 3 (8, 25 & 29) | SMC < 0.40 | 16 |
| IMI-HeartLife = Intrinsic Motivation Inventory for Heart-Healthy Lifestyle  I-CVI = item-level content validity index; EFA = exploratory factor analysis; CFA = confirmatory factor analysis; SMC = squared multiple correlation | | | | |

| Supplementary Table 4. Multi-group CFA results for measurement invariance evaluation of the IMI-HeartLife across gender (n = 703) | | | | | | | | | | | | |
| --- | --- | --- | --- | --- | --- | --- | --- | --- | --- | --- | --- | --- |
|  | Model fit | | | | |  | Model difference | | | | | |
|  | χ^2^ | df | CFI | RMSEA | SRMR |  | Δ Model | Δ χ^2^ | Δdf | ΔCFI | ΔRMSEA | ΔSRMR |
| Men (n = 359) | 382.066 | 101 | 0.939 | 0.088 | 0.063 |  | - | - | - | - | - |  |
| Women (n = 344) | 502.352 | 101 | 0.924 | 0.108 | 0.071 |  | - | - | - | - | - |  |
| Subgroup invariance (N = 703) |  |  |  |  |  |  |  |  |  |  |  |  |
| M1: Configural invariance | 884.427 | 202 | 0.931 | 0.069 | 0.063 |  | - | - | - | - | - |  |
| M2: Metric invariance | 908.542 | 215 | 0.930 | 0.068 | 0.066 |  | M2 vs. M1 | 24.115 | 13 | 0.001 | 0.001 | 0.003 |
| M3: Scalar invariance | 928.679 | 231 | 0.930 | 0.066 | 0.066 |  | M3 vs. M2 | 20.137 | 16 | 0.000 | 0.002 | 0.000 |
| CFI = comparative fit index; RMSEA = root mean square error of approximation; SRMR = standardized root mean square residual | | | | | | | | | | | | |

Supplementary Appendix

**Intrinsic Motivation Inventory for Heart-Healthy Lifestyle (IMI-HeartLife)**

※ **“Heart-healthy lifestyle”** refers to a way of living that helps prevent heart diseases such as angina and myocardial infarction. - A “heart-healthy lifestyle” comprehensively involves practicing all of the following behaviors:

• Engaging in physical activity (e.g., walking an average of 8,000 steps per day or performing moderate-intensity exercise, such as brisk walking, for 150 minutes per week)

• Maintaining healthy eating habits (e.g., eating fruits, vegetables, and whole grains daily; avoiding salty foods; not consuming sugar-sweetened beverages; and avoiding foods high in saturated or trans fats)

• Weighing yourself daily, not smoking, and avoiding binge drinking

※ Please recall your lifestyle over the past three months and respond to each item accordingly.

• Some questions may appear repetitive, but please focus only on the statement presented and respond according to your own opinion.

| No. | Item statements | Agreement | | | | | | |
| --- | --- | --- | --- | --- | --- | --- | --- | --- |
|  |  | Very  Low | |  | | | Very  High | |
| 1 | I put a lot of effort into practicing a healthy lifestyle.  나는 건강한 생활양식을 실천하기 위해 많은 노력을 하였다. | ① | ② | ③ | ④ | ⑤ | ⑥ | ⑦ |
| 2 | I think practicing a healthy lifestyle is an important activity for my heart health.  나는 건강한 생활양식을 실천하는 것이 내 심장건강에 중요한 활동이라고 생각한다. | ① | ② | ③ | ④ | ⑤ | ⑥ | ⑦ |
| 3 | I really enjoyed practicing a healthy lifestyle.  나는 건강한 생활양식을 실천하는 것이 매우 즐거웠다. | ① | ② | ③ | ④ | ⑤ | ⑥ | ⑦ |
| 4 | I think I’m good at practicing a healthy lifestyle.  나는 건강한 생활양식을 잘 실천한다고 생각한다. | ① | ② | ③ | ④ | ⑤ | ⑥ | ⑦ |
| 5 | Practicing a healthy lifestyle was fun.  건강한 생활양식의 실천은 재미있는 활동이었다. | ① | ② | ③ | ④ | ⑤ | ⑥ | ⑦ |
| 6 | I think practicing a healthy lifestyle is useful for my heart health.  나는 건강한 생활양식을 실천하는 것이 심장건강에 유용하다고 생각한다. | ① | ② | ③ | ④ | ⑤ | ⑥ | ⑦ |
| 7 | I think I practice a healthy lifestyle well compared to others.  나는 다른 사람과 비교해서, 건강한 생활양식을 잘 실천한다고 생각한다. | ① | ② | ③ | ④ | ⑤ | ⑥ | ⑦ |
| 8 | I believe practicing a healthy lifestyle is valuable to me.  건강한 생활양식을 실천하는 것은 나에게 가치가 있다고 믿는다. | ① | ② | ③ | ④ | ⑤ | ⑥ | ⑦ |
| 9 | I believe I have a choice in practicing a healthy lifestyle.  건강한 생활양식을 실천하는 것은 내가 선택하는 것이라고 믿는다. | ① | ② | ③ | ④ | ⑤ | ⑥ | ⑦ |
| 10 | I would say that practicing a healthy lifestyle was very interesting.  나는 남들에게 “건강한 생활양식을 실천하는 것은 재미있다”라고 표현할 것이다. | ① | ② | ③ | ④ | ⑤ | ⑥ | ⑦ |
| 11 | I think it’s important to practice a healthy lifestyle because it helps with heart health.  나는 건강한 생활양식을 실천하는 것이 심장건강을 향상시키는 데 도움이 될 것이라고 생각한다. | ① | ② | ③ | ④ | ⑤ | ⑥ | ⑦ |
| 12 | I thought practicing a healthy lifestyle was enjoyable.  나는 건강한 생활양식을 실천하는 것이 즐길 만하다고 생각하였다. | ① | ② | ③ | ④ | ⑤ | ⑥ | ⑦ |
| 13 | I tried very hard to practice a healthy lifestyle.  나는 건강한 생활양식을 실천하려고 열심히 시도하였다. | ① | ② | ③ | ④ | ⑤ | ⑥ | ⑦ |
| 14 | While practicing a healthy lifestyle, I felt it was very enjoyable.  나는 건강한 생활양식을 실천하면서, 무척 즐겁다고 생각하였다. | ① | ② | ③ | ④ | ⑤ | ⑥ | ⑦ |
| 15 | I think it’s important to practice a healthy lifestyle because it helps with heart health.  건강한 생활양식이 심장건강에 도움이 되기 때문에, 이를 실천하는 것이 중요하다고 생각한다. | ① | ② | ③ | ④ | ⑤ | ⑥ | ⑦ |
| 16 | I believe practicing a healthy lifestyle could be beneficial for my heart health.  나는 건강한 생활양식을 실천하는 것이 내 심장건강에 유익할 수 있다고 믿는다. | ① | ② | ③ | ④ | ⑤ | ⑥ | ⑦ |

**Scoring guideline:**

This questionnaire consists of **16 items**, each rated on a 7-point Likert scale ranging from 1 (very low) to 7 (very high) in the level of agreement. Each item reflects a positive statement related to intrinsic motivation. O**verall score** represents the mean of all 16 items. **Subscale scores** are calculated by taking the mean of the items within each subscale:

1. Interest/Enjoyment (5 items; Items 3, 5, 10, 12, 14)

2. Choice/Value/Usefulness (7 items; Items 2, 6, 8–9, 11, 15–16)

3. Competence/Effort (4 items; Items 1, 4, 7, 13)
